# The mediating role of recovery opportunities on future sickness absence from a gender- and age-sensitive perspective

**DOI:** 10.1371/journal.pone.0179657

**Published:** 2017-07-27

**Authors:** J. S. Boschman, A. Noor, J. K. Sluiter, M. Hagberg

**Affiliations:** 1 Academic Medical Center, University of Amsterdam, Department: Coronel Institute of Occupational Health, Amsterdam Public Health research institute, Amsterdam, The Netherlands; 2 Occupational and Environmental Medicine, Sahlgrenska Academy and University Hospital, University of Gothenburg, Gothenburg, Sweden; University of West London, UNITED KINGDOM

## Abstract

A lack of sufficient recovery during and after work may help to explain impaired health in the long run. We aimed to increase knowledge on the mediating role of recovery opportunities (RO) during and after work on future sickness absence from a gender- and age-sensitive perspective. We used data on RO from a Swedish national survey in 2011 and linked these to sickness absence (>14 days) two years later among the general working population (N = 7,649). Mediation of the relationship between gender and sickness absence by exposure to RO was studied through linear regression. We conducted separate analyses for RO during and after work and for three different age groups (16–29; 30–49; 50–64). The sample consisted of 3,563 men and 4,086 women. Sickness absence was higher among the women than among the men (11 days *vs* 5 days, p<0.001). Men reported statistically significantly more positive on their RO than women. RO during (ß 0.3–1.8) and after work (ß 0.4–0.6) mediated the relationship between gender and sickness absence. Mediation effects existed across age groups, with the strongest effects of RO during work found among the age group between 50 and 64 years of age (attenuation 36%). Our results indicate that gender inequality is also reflected in worse RO among women. This partially explains the increased risk of future sickness absence, particularly among those above 50 years of age. These findings show that RO during work deserve more attention in working life research.

## Introduction

Women have higher sickness absence rates than men. In the first quarter of 2016, the number of sickness absence periods among the Swedish female workforce came to 1,105,876, compared to 819,165 among the men [1]. Higher sickness absence is generally found more often among women and in older age groups [2]. Both biological sex differences and gender differences are possibly related to this increased sickness absence among women.

It is already known that women are exposed to other types and intensity of some of the work-related stressors that seem relevant for sickness absence, such as a lack of career prospects, discrimination, and sexual intimidation [3, 4]. Furthermore, adverse psychosocial working conditions, such as high mental as well as emotional demands together with low control, are more common among women [5]. It would seem plausible that women’s health can be affected by these stressful psychosocial work characteristics.

However, Smeby et al. found that the gender difference in sick leave was not reduced when working conditions, income, self-reported health and mental distress were taken into account after adjusting for age and occupation [6]. The authors conclude therefore that factors explaining the gender divide should be sought elsewhere [6].

With this study, we aimed to increase the knowledge on the mediating role of recovery opportunities during and after work and future sickness absence from a gender- and age-sensitive perspective. We formulated the following research questions: 1) Do recovery opportunities explain the relationship between gender and sickness absence two years later? And 2) Is the relationship between gender, recovery opportunities and sickness absence two years later different across age groups?

A popular viewpoint is that women, more than men, are confronted with the double burden of combining work and family, thereby reducing their recovery opportunities after work [7]. This idea is partially substantiated by findings that disrupted sleep patterns have deleterious physiological effects [8]. Furthermore, the study of Vedaa et al. [9] found that less than 11 hours sleep between shifts increased the risk for sickness absence the following month.

Although some studies suggest that poor sleep is related to absenteeism, evidence is conflicting on this aspect [5]. Evidence from longitudinal studies is scarce and insight into opportunities for recovery during and after work as a factor in maintaining health and work ability is lacking. Consequently, there is a need for expanding our knowledge, especially regarding the role of opportunities for recovery through sleep and time for relaxation and rest.

A lack of sufficient recovery may help to explain how stressful working conditions and the related acute load reactions can impair health in the long run [10]. It is therefore not surprising that recovery opportunities seem to be more important as a main predictor for work-related fatigue for example than decision latitude [11]. Recovery opportunities can be divided into two categories: 1) external opportunities for recovery, concerned with time off the job: opportunities for respite, vacation and leisure time; 2) internal opportunities for recovery, related to job design: workers’ control over rest breaks and interruptions while performing a task. Recovery after work (external recovery) is particularly necessitated when recovery opportunities during worktime (internal recovery) are insufficient [11].

## Methods

### Participants and data collection

This is a follow-up study using the 2011 Swedish Work Environment Survey on behalf of the Swedish Work Environment Authority and the 2013 Longitudinell Integrationsdatabas för Sjukförsäkrings- och Arbetsmarknadsstudier (Lisa). Linkage of both data sources was possible by means of the Swedish personal number.

The Work Environment Survey is based on the Labour Force Survey and included questions asked in a telephone interview and an additional postal questionnaire. The survey was conducted in the fourth quarter of the year and covered safety and health conditions. The development and validation of the method was described by Statistics Sweden [12]. Previous studies also reported data from this survey, for example Boström et al. [13]. We used the 2011 data.

The population eligible for the Labour Force Survey consisted of all people (aged 15–74) who were registered in Sweden. The Labour Force Survey 2011 was conducted with a sub-sample of 29,500 people. Those who answered the Labour Force Survey and were between 16 and 64 years, employed and not on long-term sick or maternity leave were invited to participate in Work Environment Survey.

The longitudinal database LISA includes all persons 15 years and older who were registered in Sweden as at 31 December each year. LISA provides detailed data on sickness, maternity and unemployment insurance for individuals by retrieving information from a number of registries. Sickness and parental data is retrieved from the Income and Tax Register and the Social Insurance Office. We used 2013 data.

This study was approved by the Regional Ethical Review Board at the University of Gothenburg, Sweden (Reg.no. 221–15).

### Measures

#### Demographic and occupational characteristics

Demographic data were retrieved from LISA and included: sex, age, educational level (categorized conform the International Standard Classification of Education [14]), marital status (categorized into ‘married or registered partner’, ‘unmarried’, ‘divorced or widowed’), and family type (in 2013, categorized into ‘children < 18yrs’ and ‘no children or >18yrs’.

#### Sickness absence

Sickness absence of more than 14 days was operationalized as the number of sickness absence days more than 14 days in 2013.

#### Recovery opportunities

Two questions on external recovery opportunities were used: ‘Do you think you get enough sleep?’ and: ‘Besides sleep, do you think you get adequate time for resting and relaxation between working days?’ Answer options for both questions were: 1) Yes, definitely enough; 2) Largely adequate; 3) No, clearly inadequate; 4) No, not adequate and 5) No, by no means adequate.

Five questions on recovery opportunities during work were used as proposed by Boström [13]. The questions ‘Can you take short breaks at any time in order to talk?’, ‘Is it possible for you to set your own work tempo?’ and ‘Does your work occasionally require you to perform nothing but repetitive tasks several times per hour?’ could be answered through the following categories: 1) Nearly all the time; 2) Roughly ¾ of the time; 3) Half of the time; 4) Roughly ¼ of the time; 5) Roughly 1/10 of the time; 6) No, not at all.

The question ‘In general, are you able to decide your working hours, within certain limits?’ could be answered through the following categories 1) Yes, I have flextime (flexible begin and end times); 2) Yes, I have relatively free working hours in another way; and 3) No, in general I cannot change my working hours.

The last question ‘Is it possible for you to decide for yourself when tasks are to be done (for example, by choosing to work a bit faster some days and taking it easier other days)?’ could be answered through the following categories 1) Always; 2) Mostly; 3) Mostly not; 4) Never.

Questions were recoded such that higher scores always reflected fewer opportunities for recovery. For questions with fewer than six categories, the score was recalculated into a weighted score so that for all questions the maximum score was six.

#### Confounding factors that influence both recovery opportunities and sickness absence

We considered marital status and having a child or children younger than 18 years of age as factors that could influence both recovery opportunities and sickness absence. Family type was operationalized in one variable with the following categories: married without children; married (registered partners) with at least one child under 18 years of age; married (registered partners) with youngest child above 18 years of age; in relationship without children; in relationship with at least one child under 18 years of age; in relationship with youngest child above 18 years of age; single mother with at least one child under 18 years of age; single mother with youngest child above 18 years of age; single father with at least one child under 18 years of age; single father with youngest child above 18 years of age; single.

We chose not to adjust for health problems, as sickness absence longer than 14 days is, by definition, absence from work as a result of illness.

#### Statistical analysis

Internal consistency of the set of questions on external recovery opportunities was assessed by means of the Spearman Brown coefficient, as this is believed to be the most suitable for two item scales [15]. Internal consistency of the set of questions on internal recovery opportunities was assessed by means of Cronbach’s Alpha. For both methods, a coefficient >0.70 was regarded as acceptable, below 0.70 as not acceptable. For the questions that showed acceptable internal consistency, we analysed the questions as a scale and summed up the scores into a total score. Normality of the scores was tested by means of visually inspecting the histogram.

We then tested for differences in sickness absence in 2013 among men and women in this study. We used an unpaired t-test to test for differences in the gross total days of sickness absence. Next, we tested for differences in recovery opportunities between men and women in this study by means of a t-test for continuous and normally distributed data or by means of a Chi-Square test for categorical data.

By using a generalized linear model, we tested external recovery opportunities and family status as covariates related to sickness absence. When family type was a statistically significant covariate, we took family type into account as potential confounder of the relationship between gender and sickness absence.

#### Mediation analysis

Mediation of the relationship between gender and sickness absence >14 days by exposure to unfavourable recovery opportunities was studied thruogh linear regression. We conducted separate analyses for internal and external recovery opportunities and for three different age groups (16–29 years of age; 30–49 years of age; 50–64 years of age).The procedure described by Hayes was used [16]. Bias-corrected bootstrap confidence intervals were constructed by using 10,000 bootstrap samples.

Preacher and Kelley’s kappa-squared was used as indication of the effect size of the indirect effect. The index is bound between 0 and 1, with a value closer to 1 representing a larger indirect effect [16]. For example, an observed indirect effect with kappa-squared = 0.20 means that the indirect effect is about 20% larger than its maximum possible value given the association between the variables observed in the sample.

The ratio of the indirect effect to the total effect was used to indicate the degree to which the effect of sex operates indirectly through recovery opportunities, in other words: the magnitude of the indirect effect. The closer this ratio is to 1, the more the effect of sex on sickness absence can be said to operate through recovery opportunities.

All analyses were conducted using the IBM SPSS 22.0 Statistical package. Statistical significance was set at p = 0.05. For the mediation analyses, we used the PROCESS macro 2.16 (available at: http://processmacro.org/download.html).

## Results

### Participants

In 2011, a total of 15,553 respondents in the Labour Force Survey telephone interview were eligible for Work environment survey. That is, in the working age (16–64) and currently working at least one hour. Of these, 12,549 participated in the Work Environment Survey telephone interview and 7,765 participants replied the Work Environment Survey postal questionnaire (response rate 62%). Those who answered the questions on recovery opportunities in the postal questionnaire were eligible for analysis (N = 7,649, 99%). Their demographics are presented in Table 1. For the analyses on internal recovery opportunities, data from 3,563 males and 4,086 females were available. For the analyses on external recovery opportunities, data from 3,534 males and 4,115 females were available. The mean age of the study population was 45 years (SD 12). About half of the population was married or a registered partner, and about two-thirds had no children under 18 years of age.

**Table 1:** Descriptives of study population, N = 7,649.

Characteristic	Men	Women
Population, N (%)	3,534 (46)	4,115 (54)
Age in years (mean, SD)	44.9 (12)	44.8 (12)
Educational level, International Standard Classification of Education (ISCED)		
Primary, N (%)	70 (2)	51 (1%)
Lower secondary, N (%)	369 (10)	394 (10%)
Upper secondary, N (%)	1 639 (46)	1 531 (37%)
Post-secondary non-tertiary, N (%)	351 (10)	239 (6%)
Short-cycle tertiary, N (%)	295 (8)	504 (12%)
Bachelor or equivalent, N (%)	371 (10)	783 (19%)
Master or equivalent, N (%)	372 (11)	560 (14%)
Doctoral or equivalent, N (%)	67 (2)	53 (1%)
Marital status		
Married or registered partner, N (%)	1,779 (50)	2,158 (52)
Never married, N (%)	1,380 (39)	1,358 (33)
Divorced or widowed, N (%)	373 (11)	596 (15)
Other, N (%)	2 (0)	3 (0)
Family type		
Children < 18yrs, N (%)	1,281 (36)	1,537 (37)
No children or >18yrs, N (%)	2,253 (64)	2,578 (63)
Internal recovery opportunties score (SD)	12.5 (4.5)	14.6 (5.0)
16–29 years of age, score (SD)	13.5 (4.6)	14.8 (4.9)
30–49 years of age, score (SD)	12.3 (4.4)	14.1 (5.0)
50–64 years of age, score (SD)	12.5 (4.6)	15.0 (5.0)
External recovery opportunities score (SD)	4.6 (1.6)	4.8 (1.7)
16–29 years of age, mean (SD)	4.5 (1.7)	4.5 (1.7)
30–49 years of age, mean (SD)	4.8 (1.6)	4.9 (1.6)
50–64 years of age, mean (SD)	4.3 (1.6)	4.7 (1.7)
On sickness absence in 2013, N (%)	239 (7)	553 (13)
16–29 years of age, N (%)	18 (4)	63 (11)
30–49 years of age, N (%)	101 (6)	241 (13)
50–64 years of age, N (%)	120 (9)	249 (15)
Number of total days with sickness absence in 2013, mean (SD)	5.4 (34)	11 (47)
16–29 years of age, mean (SD)	1.6 (11)	4.3 (24)
30–49 years of age, mean (SD)	5.2 (34)	9.7 (43)
50–64 years of age, mean (SD)	6.8 (38)	14.2 (56)

### Internal consistency recovery opportunities

The Spearman Brown coefficient for the two questions on external recovery opportunities was 0.74 and regarded as acceptable for calculating a sum score. Cronbach's Alpha for the five questions on recovery opportunities was 0.67 and 0.71 when the item on repetitive tasks was deleted. Therefore, we chose to use four questions to represent internal recovery opportunities (Table 2).

**Table 2:** Questions on recovery opportunities and their internal consistency.

Recovery opportunities	Internal consistency
**External recovery opportunities**	0.74^1^
Do you think you get enough sleep?	
Besides sleep, do you think you get adequate time for resting and relaxation between working days?	
**Internal recovery opportunities**	0.67 ^2^
In general, are you able to decide your working hours, within certain limits?	0.65 (if item deleted)^2^
In general, can you take short breaks at any time in order to talk?	0.58 (if item deleted)^2^
Is it possible for you to set your own work tempo?	0.57 (if item deleted)^2^
Is it possible for you to decide on your own when various tasks are to be done (for example, by choosing to work a bit faster some days and taking it easier other days)?	0.58 (if item deleted)^2^
Does your work occasionally require you to perform nothing but repetitive tasks several times per hour?	0.71(if item deleted)^2^

1) Spearman Brown coefficient

2) Cronbach’s Alpha

### Differences between men and women

In 2013, a total of 7% of the men and 13% of the women were on sickness absence for more than 14 days. The mean number of sickness absence days more than 14 days was 5.4 (SD 34) for the men and 11 (SD 47) for the women. Both the proportion of persons (p<0.01) and the mean number of days on sickness absence (p<0.01) were statistically significant higher among the women.

For both the internal and external recovery opportunities, we found that men reported statistically significantly more positive on their opportunities for recovery than women (see Table 1). We did not find that family type was statistically significantly related to sickness absence for either men (p = 0.57) or women (p = 0.42).

### Mediating effect of external recovery opportunities

External recovery opportunities statistically significantly mediated the effect of gender on sickness absence days (Table 3). The indirect effect of gender on sickness absence through recovery opportunities is around 0.5% of its maximum possible value.

**Table 3:** Linear regression of number of sickness absence days (>14 days) explained by gender with recovery opportunities after work as potential mediator.

	Direct effect Gender		Indirect effect		Effect size	Total effect Gender+ external recovery opportunities		
	Effect	95%CI	Effect	95%CI	Kappa-squared %	Attenuation %	Effect	95%CI (bootstrap)
Total population (n = 7,649)	**5.0**	3.16–7.25	**0.4**	0.21–0.64	0.5	8	**5.4**	3.55–7.25
Age group 16–29 (n = 997)	**2.7**	0.30–5.07	0.01	-0.33–0.42	n.s.	n.s.	**2.7**	0.29–5.10
Age group 30–49 (n = 3,613)	**4.3**	1.74–6.78	**0.3**	0.02–0.62	0.3	5	**4.5**	2.00–7.05
Age group 50–64 (n = 3,039)	**6.8**	3.30–10.25	**0.6**	0.15–1.26	0.7	9	**7.4**	3.95–10.87

Bold printed figures represent statistically significant findings. n.s. = not statistically significant

External recovery opportunities were also a statistically significant mediator among the age groups 30–49 and 50–64. The indirect effects were found to be less than 1% of its maximum possible value (kappa-squared). The highest statistical significant indirect effect relative to the total effect was found for the women 50–64 years. Among these women, 9% of the effect of gender on sickness absence operated through external recovery opportunities (attenuation).

### Mediating effect of internal recovery opportunities

Internal recovery opportunities statistically significantly mediated the effect of gender on sickness absence days, for both the total group and across all age categories (Table 4). The indirect effect on sickness absence through internal recovery opportunities ranged from about 1% to 8% of the maximum possible value (kappa-squared). The highest statistical significant indirect effects were found for the women of 50–64 years. For these women, 36% of the effect of gender on sickness absence operated through internal recovery opportunities (attenuation).

**Table 4:** Linear regression of number of sickness absence days (>14 days) explained by gender, with internal recovery opportunities as potential mediator.

	Direct effect Gender		Indirect effect		Effect size	Total effect Gender+ external recovery opportunities		
	Effect	95%CI	Effect	95%CI	Kappa-squared %	Attenuation %	Effect	95%CI (bootstrap)
Total population (n = 7,649)	**4.2**	2.36–6.09	**1.1**	0.67–1.53	1.3	26	**5.3**	3.48–7.14
Age group 16–29 (n = 991)	**2.2**	-0.23–4.62	**0.3**	0.03–0.98	8.7	14	**2.5**	0.13–4.94
Age group 30–49 (n = 3,598)	**4.2**	1.70–6.61	**0.8**	0.32–1.33	1.0	17	**4.9**	2.51–7.34
Age group 50–64 (n = 3,039)	**5.0**	1.36–8.53	**1.8**	0.92–2.90	1.8	36	**6.8**	3.31–10.26

Bold printed figures represent statistically significant findings.

## Discussion

### Findings

Women have fewer recovery opportunities during and after work compared to men. The increased risk for future sickness absence two years later is mediated for a small part by both internal and external opportunities for recovery. These mediation effects of recovery opportunities exist across age groups, with the strongest effect of both internal and external recovery opportunities among the age group between 50 and 64 years of age. However, the measures for effect size showed that only a very small part of the effect of gender on sickness absence operates through external recovery opportunities and a somewhat larger part through internal recovery opportunities.

### Reflections on recovery opportunities

In this study, we chose a way of operationalizing recovery opportunities similar to that used by Boström et al. [13]. However, we did not look at the different aspects of recovery opportunities separately as was done in that study. We chose to combine the answers to the questions and conduct analyses in which the concepts of ‘internal recovery opportunities’ and ‘external recovery opportunities’ are captured reasonably well. We acknowledge that some aspects of these opportunities were not available in our dataset. For example, we had not data available on opportunities to choose when to go on holiday, to have days off, or the impact of irregular working hours on one’s private life [11]. Therefore, our scores that represent recovery opportunities could be regarded as non-optimal. A prospective study design in which the Recovery Opportunities Scale [11] is used as measurement instrument would be optimal.

Another point that is worth discussing is that we chose a different strategy to operationalize external recovery opportunities than Van Veldhoven and Sluiter did in their Recovery Opportunities Scale [11]. In this scale, the opportunities to have enough time for rest and relaxation play a central role. We now chose to study how people experience their external recovery opportunities: do they experience that they get enough sleep and rest between working days? Our rationale for looking at recovery opportunities this way is that having opportunities for recovery outside work does not necessarily mean that these opportunities lead to rest and relaxation. A demanding family life could be an important factor in disrupting this [17].

The increasing partially mediating effect of both internal and external recovery opportunities with increasing age can be explained in various ways. First, it is possible that the effect of a lack of recovery opportunities might become stronger for those of a higher age with health problems. This idea is comparable to the finding that work demands relate differently to a high need for recovery across the lifetime [18]. Second, it is possible that older workers suffer from health problems that are different from their younger colleagues, for which the mediating effect is stronger.

### Comparison to other studies on sickness absence

Findings regarding the relationship between family characteristics and sickness absence are conflicting. Over the years, some authors did not find such a relationship [2, 19], whereas others did [20]. We did not find an effect of family type on sickness absence. Additionally, Allebeck and Mastekaasa argued that there is insufficient scientific evidence for an effect of marital status on sickness absence, and that similarly, there is insufficient or weak scientific evidence regarding children living at home [2] or work-family interference [21]. However, other studies point in a different direction. Floderus et al. [20] found that parenthood increased the likelihood of sickness absence, particularly in young women and in single women. Although we cannot rule out that our results on recovery opportunities are not confounded by aspects of family structure, we believe that this is unlikely. In our study, the mediation effects increased with increasing age. This finding is not in line with the parenthood hypothesis for explaining increased sickness absence among women.

### Strengths and weaknesses

A strong aspect of our study is the longitudinal design that allowed us to study the mediating effect of recovery opportunities on sickness absence two years later. Although we cannot make any firm conclusions about causality, we believe that this type of evidence is superior to cross-sectional research findings and allows for more comprehensive conclusions. Our findings add to the current knowledge on the prevention of long-lasting (work-related) health problems. Our results substantiate the idea that interventions focusing on improving internal recovery opportunities [22, 23] could be potentially effective.

In this study, we focused on age-specific mediating effects of recovery opportunities on sickness absence. We acknowledge that other approaches in studying mediating factors for sickness absence could also be of value. Another weakness of our study is that by using the Swedish Work Environment Survey, some forms of work, e.g. hazardous work and temporary employment relations were underrepresented in the study sample. These groups are known to be difficult to reach in the Work Environment Survey [24]. On the other hand, we believe that generalizability of our study results is still sufficiently broad, as our data is based on the general working population in Sweden.

Our findings might not be generalizable to the situation in other countries due to the Swedish context. Sweden is considered to be a relatively advanced welfare state with a high level of social security. Among the benefits are for example family benefits: workers are able to be home from work to take care of their children for up to 480 days per child. Those who are ill or disabled and can’t work receive benefits as well. In situations where benefits are lacking, minimal or insufficient, differences in sickness absence and recovery opportunities between men and women are likely to be larger.

### Practical implications

This study points out that there are differences in recovery opportunities between men and women. In Sweden, approximately 90% of the workforce display typical gender segregation, e.g. men and women have different jobs, different employers, and different tasks, and are working in separate branches. Although the Nordic countries are historically known for their policies and commitment to gender equity, this seems to have been unsuccessful and there is a considerable gender-based occupational segregation and inequality [25, 26]. Our results indicate that this inequality is also reflected in worse internal recovery opportunities among women. This aspect deserves attention when aiming for gender equity in working life.
